# Cationic Polymethacrylate-Modified Liposomes Significantly Enhanced Doxorubicin Delivery and Antitumor Activity

**DOI:** 10.1038/srep43036

**Published:** 2017-02-22

**Authors:** Wenxi Wang, Anna Shao, Nan Zhang, Jinzhang Fang, Jennifer Jin Ruan, Benfang Helen Ruan

**Affiliations:** 1 College of Pharmaceutical Science, Zhejiang University of Technology, Hangzhou, China

## Abstract

Liposome (LP) encapsulation of doxorubicin (DOX) is a clinically validated method for cancer drug delivery, but its cellular uptake is actually lower than the free DOX. Therefore, we modified DOX-LP with a cationic polymer (Eudragit RL100; ER) to improve its cellular uptake and antitumor activity. The resulting DOX-ERLP was a 190 nm nanoparticle that was absorbed efficiently and caused cancer cell death in 5 hrs. Growth as measured by the MTT assay or microscopic imaging demonstrated that DOX-ERLP has at least a two-fold greater potency than the free DOX in inhibiting the growth of a DOX resistant (MCF7/adr) cell and an aggressive liver cancer H22 cell. Further, its *in vivo* efficacy was tested in H22-bearing mice, where four injections of DOX-ERLP reduced the tumor growth by more than 60% and caused an average of 60% tumor necrosis, which was significantly better than the DOX and DOX-LP treated groups. Our work represents the first use of polymethacrylate derivatives for DOX liposomal delivery, demonstrating the great potential of cationic polymethacrylate modified liposomes for improving cancer drug delivery.

Liposomes are drug delivery vehicles, offering temporal control of drug release and site-specific drug delivery for a wide range of drugs with different physiochemical properties^1^,^2^. For example, Doxorubicin Hydrochloride (DOX) is a DNA intercalator which has a broad-spectrum of anti-tumor activity, including the clinical treatment of acute leukemia, malignant lymphoma, breast cancer, bladder cancer and so on^3^. However, its side effects such as cardiac damage and bone marrow suppression can seriously limit its clinical application. Encapsulation of DOX with liposomes was an improvement that enabled changes in its *in vivo* distribution, increased its anti-tumor effect, reduced its cardiac toxicity, and allowed it to become a welcome product on the market^4^,^5^. However, liposomes have limitations, including poor stability, drug leakage, short residence time, and inadequate dispersion. To overcome these problems, multiple research groups have tried to modify drug-carrying liposomes using various polymeric materials to achieve favorable effects^6^. For example, Polyvinyl alcohol modification improved the physical stability of the liposome membrane^7^,^8^. Coating a liposome carrying a peptide-drug with a hydrophobic modified dextran greatly stabilized the drug and increased its elimination half-life^9^. Poly(N-isopropylacrylamide-co-acrylamide) modified liposomes^10^ and negative charged gangliosides^11^ were also used to reduce drug leakage and to improve the physical stability of liposomes during the storage period.

In addition, coating with hydrophilic polymers prevented liposomes from being adsorbed to plasma proteins and opsonins and from being phagocytosed by macrophages; this extended the *in vivo* circulation time of liposomes in blood, increased the drug distribution in tissues and organs outside the reticuloendothelial system and strengthened the drug’s targeting properties^12^,^13^. Hydrophobically modified chitosan-coated liposomes improved the adhesion of the liposomes and prolonged its retention time on the mucous membrane for better absorption^14^,^15^.

Further, the gH625 peptide modification provided DOX liposomes with targeted drug delivery and greatly overcame DOX resistance in lung adenocarcinoma cell lines^16^. CXCR4-antagonist peptide R-liposomes efficiently inhibited CXCR4-dependent migration and significantly reduced cancer metastases^17^. Stealth liposome encapsulation provided neurological drugs with the ability to pass the blood brain barrier^18^. Also, poly(ε-caprolactone)-b-poly(N-vinylpyrrolidine) was used to make micelles to enhance the antitumor effect of DOX in lymphoma^19^.

Polymethacrylate has been widely used in pharmaceutical preparations to achieve controlled release in tablets, but was only recently used in liposome modification. Eudragit EPO (containing 1:2:1 ratio of butyl-, dimethyl aminyl ethyl-, methyl polymethacrylate) was used to modify acyclovir and minoxidil liposomes, and found that it significantly improved the stability of the liposomes and enhanced the percutaneous penetration of the drug^20^. Eudragit S100 and Eudragit L100 (neutral methyl, ethyl polymethacrylate, respectively) were used to coat atenolol liposomes to improve encapsulation efficiency and mucous membrane adhesion^21^. The amino-bisphosphonate Zoledronic acid (ZOL) has potent anticancer activity and its encapsulation into a stealth liposome formulation reduced the binding of ZOL to bone and increased its bioavailability in extraskeletal tumor sites through the enhanced permeability retention (EPR) effect^22^.

Recently, researchers have shown that although DOX-LP has improved anti-tumor effects, much less DOX was absorbed into the cells from DOX-LP than from the free DOX; the enhanced anti-tumor effect is mainly due to the Enhanced Permeation Retention (EPR) effect^23^,^24^. EPR effect occurs when nano-sized agents with long circulation times preferentially move into the tumor tissue through leaky tumor vasculature and are retained in the tumor bed through reduced lymphatic drainage^25^,^26^. To improve the *in vivo* efficacy of DOX, we modified the DOX-bearing liposomes with cationic polymethacrylate Eudragit RL100, which contains positively charged quaternary ammonium groups, because cationic polymers should provide better affinity to certain drugs, cell membrane and mucousa through electrostatic interactions.

Reported herein are the preparation and characterization of the Eudragit RL100 modified DOX-bearing liposomes. The new formulation showed a slow DOX release from the liposomes and a high DOX uptake by the cells, and resulted in significantly improved antitumor activities in various cancer cells and in an animal model for cancer.

## Materials and methods

### Materials

Phosphatidylcholine from soybean (95%) was purchased from Lipoid GmbH (Ludwigshafen, Germany), Eudragit^®^ RL100 was obtained from Evonik Industries AG (Darmstadt, Germany), and doxorubicin (DOX) was obtained gratis from Zhejiang Hisun Pharmaceutical Co., Ltd. (Taizhou, China). RPMI 1640 medium was purchased from M&C Gene Technology Inc. (Beijing, China). Trypsin and EDTA were purchased from Amresco (Solon, OH, USA). Fetal bovine serum (FBS) was purchased from Zhejiang Tianhang Biological Technology Co., Ltd. (Zhejiang, China). H22, MCF7 and MCF7/adr (DOX resistant cell line) were purchased from Chinese Academic of Science (Shanghai, China). ICR mice were purchased from Zhejiang Institute of Medical Science (Hangzhou, CN). The animal experiments were carried out at the animal facility of Zhejiang No. 1 Hospital, and permission was obtained from Zhejiang Province Health Planning Committee of the subject of animal experiments with accreditation number of SYKX (Zhe) 2013-0180.

### Preparation and characterization of DOX-loaded Eudragit RL 100 Liposome (ERLP)

ERLP was prepared by the solvent evaporation method and DOX was loaded by the (NH_4_)_2_SO_4_ gradient method^27^. Eudragit RL100 (200 mg), phosphatidylcholine (200 mg) and cholesterol (50 mg) were mixed and dissolved in absolute ethanol (15 ml) by heating and sonication, and then 0.2 M (NH_4_)_2_SO_4_ solution (15 ml) was added dropwise. The organic solvents were evaporated under magnetic stirring at 55 °C for 4 h, sonicated for 5 sec each for 40 cycles at 400 watts, and the resulting suspension was dialyzed in saline (200-fold volumes) for 24 h to remove the free (NH_4_)_2_SO_4_. The resulting ERLP was incubated with DOX solution (6 mg/ml) at 60 °C for 0.5 h to obtain DOX-ERLP.

DOX-loaded LP was prepared in a similar method as above, except for no addition of Eudragit RL100.

### Diameter and particle size distribution of DOX-ERLP

The diameter and particle size distribution of liposomes, such as Z-average diameter (Zavd), Polydispersity index (PdI), Intesity-mean diameter (Imd), Volume-mean diameter (Vmd), Number-mean diameter (Nmd), were measured by photon correlation spectroscopy (PCS) on a Malvern Zetasizer nano ZS (Malvern instruments, UK). The surface charge was estimated by measuring the zeta potential (ZP) based on the electrophoretic mobility without dilution.

### Determination of DOX-encapsulating efficiency by ultracentrifugation

The encapsulation efficiency (EE) of DOX in DOX-LP and DOX-ERLP was measured by an ultracentrifugation test^28^. The liposomes were ultracentrifuged at 197, 000 × g below 4 °C for 4 h to pellet the liposomes. DOX in the supernatant was quantified by UV spectrophotometry at the wavelength of 495 nm, and total DOX in the liposomes was determined after liposomes were dissolved in 80% ethanol containing 0.1 M HCl. EE was calculated according to the following equation (1):


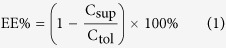


C_sup_ is the concentration of DOX in the supernatant and C_*tol*_ is the total concentration of DOX.

### *In vitro* drug release

Solutions (1 ml) of free DOX, DOX-LP and DOX-ERLP were transferred to an individual dialysis bag, dialyzed in phosphate buffer solution (PBS, 200 ml, pH 7.4), and shaken (50 rpm) at 37 °C. Aliquots (5.0 ml) were taken from the released medium at 0.5, 1, 2, 4, 6, 8, 12, 20, 30, 38, 48, 60, 72 hours, and the same aliquot of blank PBS was added back to keep volume constant. The aliquoted samples were diluted and treated with 0.1 M HCl in 80% ethanol (2 volumes) to determine the DOX fluorescence intensity (Ex = 480 nm, Em = 590 nm). The DOX concentration was calculated based on the standard curve, and the accumulated release percentage was calculated based on the equation (2) below:


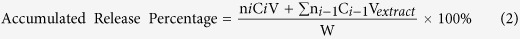


In this equation, n_*i*_ is the fold of dilution; C_*i*_ is the DOX concentration in each sample; V means the medium volume (200 ml); V_*extract*_ is sample volume (5.0 ml); and W represents the total amount of DOX.

### Fluorescence microscopic observation of cellular uptake of DOX liposomes by MCF-7

Breast cancer cell line MCF-7 was seeded in a 96-well plate (1 × 10^4^/well) in RPMI-1640 medium with 10% fetal bovine serum and grown in 5% CO_2_ incubator at 37 °C for 24 hours. After removing the old medium, the cells were treated with free DOX, DOX-LP or DOX-ERLP with final DOX (5 μg/ml) in fresh medium for 5 hours. Then, the culture media was removed; the cells afterwards were washed with cold PBS once, treated with 8 g/ml Hoechst 33342 in RPMI-1640 (200 μl) media and incubated for 0.5 hours. After removing the culture media, cells were washed twice with cold PBS, and cell viability (EX350 nm, EM 460 nm) and DOX uptake (EX 480 nm, EM 590 nm) were observed at two different corresponding wavelengths using a fluorescence microscope.

### Kinetic study of DOX cellular uptake

Attached MCF-7 cells (5 × 10^4^/well) in 48-well plate were grown overnight to reach 70% ~ 80% confluence, and then treated with DOX or DOX-ERLP (300 μl; final 5 μg/ml DOX). After incubation for 15 min, 1 hour, 2 hours, 4 hours, 6 hours, 8 hours, 12 hours, or 16 hours, the cells were harvested by removing the medium, washed twice with cold PBS, and processed according to the method described previously^19^. The harvested cells were lysed in 1% Triton X-100 and 0.1% SDS lysis solution (300 μl), and aliquots (100 μl) were taken and mixed with acetonitrile (200 μl) by vortexing to extract DOX. After centrifugation at 12,000 rpm for 20 min, aliquots (20 μl) were taken from the DOX containing CH_3_CN layer for HPLC analysis. Samples (20 μl) were injected into a HPLC system equipped with a fluorescent detector (EX 480 nm, EM 590 nm) and a Hypersil ODS (C18) column which was developed using a solvent mixture of CH_3_CN and 50 mM NaH_2_PO_4_ (pH 2.8; 70:30 ratio) at 40 °C. In addition, aliquots (20 μl) from the cell lysates were taken for protein quantification by the Bradford assay. The cellular DOX uptake was presented as a ratio of DOX concentration to protein concentration.

### Flow cytometry analysis of cellular uptake of DOX liposomes by H22 cells

Suspension cells (H22 liver cancer cells; 8 × 10^5^ cell/ml), grown in 30 ml RPMI with 10% FBS, were treated with DOX, DOX-LP or DOX-ERLP (final 5 μg/ml DOX). After incubation for 12 hours, cells were subjected to FACS analysis using BD FACS Calibur. DOX fluorescence associated with cells was measured using FL2 channel at EX 480 nm and EM 590 nm. For each sample, 2 × 10^4^ events were acquired, and analysis was carried out by triplicate determination on at least three separate experiments. The percentage of fluorescent cells and their fluorescent strength were quantified.

For further confirmation of the DOX uptake, the cells were harvested by removing the medium, and 50% pellets were washed twice with cold PBS. The harvested cells (washed/unwashed) were lysed in 1% Triton X-100 and 0.1% SDS lysis solution (300 μl) for DOX fluorescent measurement at EX 480 nm and EM 590 nm.

Aliquots of the recovered media were treated with 0.1 M HCl in 80% ethanol (2 volumes) to release the DOX from liposomes and determine DOX level by fluorescence and HPLC method.

### *In vitro* antiproliferation assay

Cells (H22 or MCF-7/adr; 10^3^/well) in 96-well plates were pre-incubated in RPMI media with 10% FBS at 37 °C with 5% CO_2_, then treated with a series of dilutions of DOX, DOX-LP or DOX-ERLP (0–50 μg/ml). The cell growth was observed under a microscope and by MTT assay. The MTT assay was performed by removing the old media and treating the cells with MTT (5 mg/ml; 20 μl) in RPMI1640 medium without phenol red (180 μl) for 4 h. After removing the MTT reagents, the resulting blue formazan in cells was dissolved in DMSO (150 μl) and measured by Microplate Reader at 570 nm to calculate the cell growth inhibition (IC_50_).

### *In vivo* antitumor efficacy

H22 cells were inoculated into the abdomen of ICR mice and grown for a week^29^. The ascites were extracted and diluted to 10^7^ cells per ml, and an aliquot (0.2 ml) was hypodermically injected (*ih*) at the right axilla of each ICR mouse. Tumor gobbets of approximately 100 mm^3^ in volume were observed in 4 days, and mice were randomly divided into 4 groups with 8 mice in each group: normal saline (Group A), Free DOX (Group B), DOX-LP (Group C) or DOX-ERLP (Group D). The dose (5 mg/kg DOX) was administered by tail intravenous injection (*iv*) daily, and the maximum diameter (a) and the minimum diameter (b) of tumors were also measured with a vernier caliper to calculate the tumor volume based on equation (3). On the twelfth day, the mice were sacrificed by cervical dislocation, and tumor tissues were removed, precisely weighed and fixed in 10% formalin for further tumor pathology characterization by routine hematoxylin and eosin (HE) staining methods. All methods were performed in accordance with the relevant guidelines and regulations.


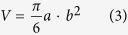


### Statistical analysis

Statistical analyses of the samples from DOX-LP and DOX-ERLP were performed using a one-way analysis of variance (ANOVA) with Neumann-Keul’s multiple comparison test or Kolmogorov-Smirnov where appropriate using the Excel software, and P-values < 0.05 were considered statistically significant. All data are reported as the mean ± the standard deviation (SD) unless otherwise stated.

## Results and Discussion

### Preparation, optimization and characterization of DOX-loaded ERLP

DOX-ERLP was prepared by soaking DOX into the ERLP liposome prepared using a (NH_4_)_2_SO_4_ gradient method. The optimal ratio of ER and LP for ERLP liposome preparation was determined based on parameters such as the particle size, size distribution, electrical potential, and most importantly the stability of the liposomes. As shown in Table 1, when a very small amount of ER was used, the prepared ERLP showed large particle size, wide distribution, and large dispersion index. When the ER:LP ratio was increased to greater than 1/3, the prepared ERLP showed obviously smaller particle size, narrow distribution, and small dispersion index. Perhaps the positively charged ER changed LP distribution in ethanol/water solvent system, so ERLP was precipitated more uniformly. Also, because ER is positively charged, the surface potential of the ERLP increased dramatically when higher amount of ER was added; the increased Zeta potential may reduce aggregation and precipitation of the particles, so smaller particles can be obtained when more ER was added. We used the 1:1 ratio of RL:LP to prepare the ERLP for biological function analyses, because the liposome has suitable particle size and nice stability.

**Figure 1:**
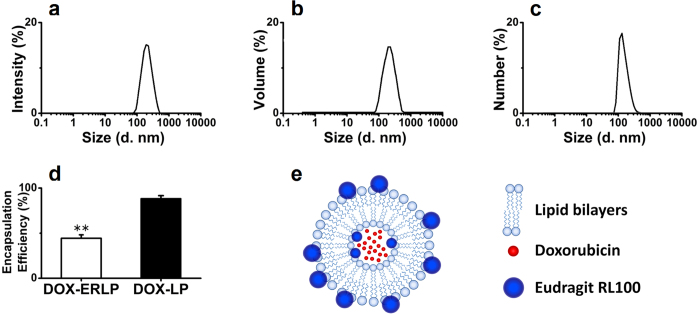
ERLP and the encapsulation of DOX-ERLP. Size distribution of ERLP (1:1 ratio) by intensity (**a**), volume (**b**), and numbers (**c**). (**d**) DOX encapsulation was measured by ultracentrifugation followed by UV measurement at 495 nm. The difference in encapsulation efficiency between DOX-ERLP and DOX-LP is significant with a p-value < 0.01. (**e**) Possible structure of ERLP.

The resulting ERLP (1:1 ratio) showed a Zeta average size of 189.5 nm and the poly-dispersion index (PdI) of 0.127 (shown in Fig. 1a–c). The Zeta potential of ERLP was +40.1 mV, which is much higher than that of DOX-LP (−2.20 mV). As shown in Fig. 1d, the encapsulation efficiency (EE) of DOX-ERLP was 44% (with 4.3% loaded drug), which is lower than the DOX-LP (EE 88%). Perhaps the polymethylacrylate modification reduced the permeability of DOX through the lipid membrane. Also, the (NH_4_)_2_SO_4_ concentration was found to be critical for the encapsulation efficiency; 0.2 M (NH_4_)_2_SO_4_ was the optimal concentration which gave the highest EE, whereas 3 M (NH_4_)_2_SO_4_ showed no improved EE and caused large particle precipitation maybe due to the accelerated precipitation rate of polymethylacrylate.

In summary, the possible structure of the ERLP liposome (Fig. 1e) is that LP forms a membrane double layer, and ER is surrounded by the phosphate groups of LP and distributed at both the outside and the inside surface, because ER is a polymethacrylate containing positively charged functional groups which was expected to interact with the negative charged phosphate group of phospholipids.

### Drug release *in vitro*

**Figure 2:**
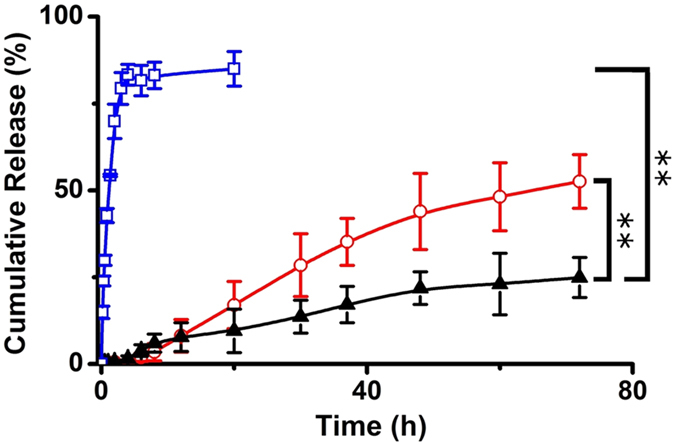
***In vitro*** **DOX release profile of Free DOX (□), DOX-LP(⚪) and DOX-ERLP(▲).** Significantly reduced DOX release in DOX-ERLP formulation was observed in comparison with the free DOX and DOX-LP with p values < 0.01.

Figure 2 showed DOX release from the dialysis bag containing free DOX, DOX-LP and DOX-ERLP. Free DOX released rapidly and completed within 4 h; DOX-LP significantly slowed the DOX release and only 52% DOX was released in 72 hours. Interestingly, DOX-ERLP demonstrated even smaller amount of DOX release and only 25% released in 72 hours. This indicated that the cationic polymethylacrylate modifications further reduced the release of drugs in liposomes and demonstrated a better, sustained release performance.

### DOX-ERLP demonstrated better cellular uptake and growth inhibition activities in MCF7 cells than free DOX and DOX-LP using fluorescent assays

DOX has strong red fluorescence (EX 550 nm and EM 620 nm), so we directly analyzed DOX cellular uptake using the fluorescence microscope. Hoechst 33342 can penetrate the cell membrane and bind to DNA, so the nucleus of live cells can be stained to show strong blue fluorescent (EX 350 nm, EM 461 nm). Therefore, we used Hoechst 33342 to locate the cell nucleus and detect cell death. At the same time, we investigated the cellular uptake and distribution of DOX using the fluorescence of DOX itself. For further evaluation of the cellular uptake, we extracted the DOX from cells at various time points and analyzed by reversed-phase HPLC with fluorescence detection.

**Figure 3:**
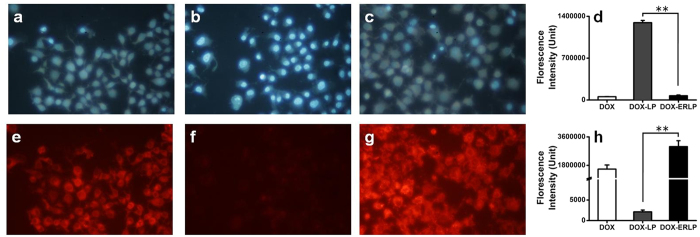
Cell viability and DOX uptake analyses after treating the MCF-7 cells with free DOX (**a**,**e**), DOX-LP (**b**,**f**) and DOX-ERLP (**c**,**g**) (final 5 μg/ml DOX) for 5 hours. (**d**) Cell viability analysis by Hoechst33342 (**a**,**b**,**c**,**d**; EX 350 nm and EM 460 nm). (**h**) DOX uptakes analysis (**e**,**f**,**g**,**h**; EX 480 nm and EM 590 nm). p-Values (<0.01**) were obtained after comparing the results between the DOX-LP and DOX-ERLP samples.

After being cultured with free DOX, DOX-LP or DOX-ERLP (5 μg/ml DOX) for 5hrs, the MCF-7 breast cancer cells were stained with Hoechst 33342 and observed with the fluorescence microscope (Fig. 3). As shown in Fig. 3a and e, free DOX showed significant amounts of cellular uptake and caused cell death. However, within a short amount of incubation time, DOX-LP (Fig. 3b,f) was not absorbed in significant amounts and no cell death was observed. In contrast, DOX-ERLP (Fig. 3c,g) showed much more significant absorbance than the free DOX by MCF-7 cells, and greater cell death was observed.

Figure 3d and h compares the difference in the cell viability and DOX uptake among the three versons of DOX formulation after a short term treatment. The lowered DOX uptake in DOX-LP treated cells is in agreement with the early report that liposomes significantly decreased the immediate uptake of DOX by Eliaz *et al*.^21^,^23^. Importantly, liposome modified by polymethylacrylate (ERLP) sharply increased the DOX in the cells. These results showed that DOX-ERLP had greater penetration through the cell membrane than DOX-LP, resulting in improved antitumor activity.

### DOX-ERLP demonstrated superior cellular uptake by H22 cells in suspension than free DOX and DOX-LP by fluorescence and flow cytometry analyses

**Figure 4:**
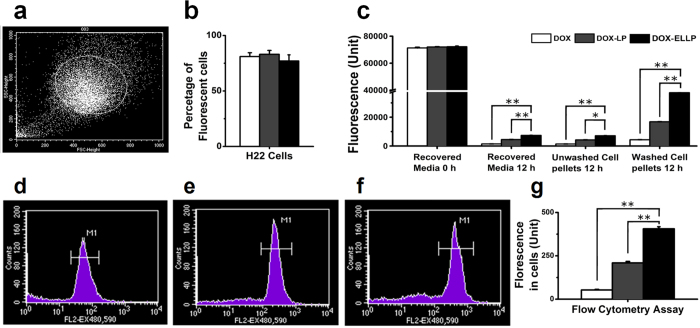
DOX uptake analyses. After H22 cells in suspensions were treated with (**d**) free DOX, (**e**) DOX-ERLP and (**f**) DOX-LP (5 μg/ml DOX) final for 12 hours, the distribution of DOX fluorescence was analyzed by flow cytometry (EX 480 nm and EM 590 nm). (**a**) The population of normal size cells selected for fluorescent analysis. (**b**) Percentage of fluorescent cells in total amount of cells. (**c**) Distribution of DOX in cells and media were analyzed by fluorescent HPLC analyses (EX 480 nm and EM 590 nm). The fresh DOX media samples (0 h) were also analyzed under the same condition to serve as controls for extraction efficiency. FACS analysis of fluorescent intensity in cells treated with (**d**) free DOX, (**e**) DOX-LP and (**f**) DOX-ERLP. (**g**) Comparison of DOX uptakes in the free DOX and DOX-LP treated samples by FACS analysis (**d**,**e**,**f**,**g**; EX 480 nm and EM 590 nm). p-Value (<0.01**; <0.05*) were obtained after comparing the results between the DOX-ERLP and DOX-LP or free DOX samples.

Free DOX, DOX-LP or DOX-ERLP uptakes were also measured in a sensitive suspension cell line (H22; 5 μg/ml DOX final concentration), because the suspension cells might have better exposure to the liposomes than the attached cells (MCF7). After a longer incubation of 12 hours, DOX uptakes were measured using FACS analysis as shown in Fig. 4a,c,d,e, and also by fluorescent measurement after DOX extraction (Fig. 4b). Both experimental results demonstrated that uptake of the DOX-ERLP was 2–fold more than that of the DOX-LP and 8–fold more than that of the free DOX; this further confirmed that addition of Eudragit RL100 can significantly enhance the cellular uptake of the DOX-loaded liposomes. Interestingly, DOX-LP showed much higher fluorescence in H22 cells after 12 hours incubation than in the MCF7 cells after 5 hours incubation; perhaps the cells in suspension can interact with the DOX-LP more efficiently than the attached cells, and also a longer incubation time might be able to overcome the slow cellular uptake problem with DOX-LP.

Interestingly, even though the cells were treated with the same amount of DOX and processed under the exactly the same conditions, repeated experiments showed that after 12–15 hours incubation, the fluorescence levels were consistently higher in DOX-ERLP samples, intermediate in DOX-LP samples and low in free DOX samples, regardless of whether it was a cell fraction or a media fraction (Fig. 4b and Table 2). Why was there a significant loss of DOX in the free DOX treated cells and its media (Table 2)? This was less likely to be an issue with the extraction, because all experiments were done under the same conditions, and extraction efficiency was measured using fresh (0 h time point) free DOX, DOX-LP and DOX-ERLP reagents and 100% extraction was obtained (Table 2). We hypothesized that the cellular metabolism quickly reduced the amount of DOX in free DOX treated samples after 12 hour treatment. Since DOX-ERLP showed the lowest DOX release (Fig. 2) and the highest cellular DOX uptake (Fig. 3), it would be reasonable to observe higher residual levels of DOX in DOX-ERLP treated samples than those in free DOX or DOX-LP.

### DOX-ERLP eNnhanced the growth inhibition of a DOX resistant strain (MCF-7/adr)

To investigate if DOX-ERLP has improved anti-proliferation activity towards DOX resistant cancer cell lines, MCF-7/adr cells were treated with three versions of DOX formulations to investigate their cytotoxicity and the cellular metabolism of free DOX.

**Figure 5:**
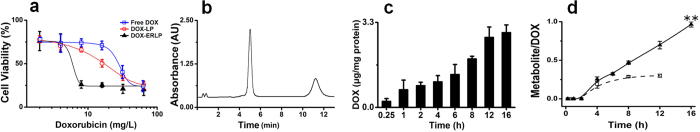
Growth inhibition and DOX metabolite formation. (**a**) Growth inhibition of a DOX resistant cell line (MCF-7/adr) by free DOX, DOX-LP and DOX-ERLP with a p value < 0.05. (**b**) HPLC analysis of cellular metabolites of DOX; t_R_ 4.5 min (DOX), t_R_ 11.2 min (DOX metabolites). (**c**) Time course of cellular DOX in DOX-ERLP treated samples. (**d**) Time course of the ratio between the metabolite (t_R_ 11 min) and cellular DOX in MCF-7/adr cells treated with Free DOX (◽) and DOX-ERLP (▴). The ratio difference was significant with a p value < 0.01 (**).

As is shown in Fig. 5a and Table 3, DOX-ERLP demonstrated at least 2-fold higher growth inhibition than free DOX (P < 0.05). Also interestingly, in the HPLC analysis (Fig. 5b) of cellular extracts of DOX, a new peak at retention time (t_R_) of 11.2 min was observed. In addition, fluorescent HPLC analysis showed that both the cellular DOX level (Fig. 5c) and the relative amount of the 11.2 min peak to the DOX peak increased (Fig. 5d) with prolonged incubation time; this peak was not found in the DOX storage solution, which suggests that the new peak might be the cellular metabolite of DOX. In addition, in the MCF-7/adr cells, the 11.2 min peak increased faster in DOX-ERLP treated cells than in the free DOX treated one. Unfortunately, we are unable to obtain mass spectrometry data for this metabolite. Therefore, the role of the metabolite (11.2 min) and its potential antitumor activity remain to be investigated. However, the increased level of the DOX metabolite was an indication that DOX can be metabolized in cells, which supported early observation of lossing DOX in free DOX treated samples after prolonged incubation with cells (Table 2).

### DOX-ERLP enhanced growth inhibition of an aggressive liver cancer cell H22

**Figure 6:**
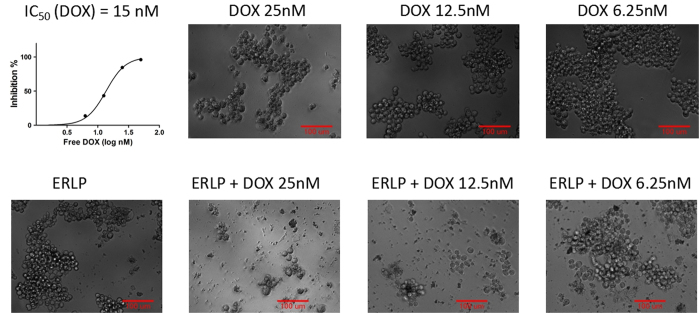
Growth inhibition of H22 liver cancer cells by free DOX (upper panel) and DOX-ERLP (lower panel). Significantly (2-fold) enhanced growth inhibition was observed with DOX-ERLP.

Improved anti-proliferative activity of DOX-ERLP on H22 cells was also observed. As shown in Fig. 6 upper panel, DOX demonstrated potent inhibition of liver cancer cell line (H22) with an IC_50_ of 15 nM for free DOX. Interestingly, in the presence of ERLP, nearly complete growth inhibition of H22 cells was observed at 12.5 nM DOX-ERLP; the estimated IC_50_ of DOX-ERLP was lowered by at least 2-fold (Fig. 5 lower panel). A comparison of DOX-ERLP enhanced anticancer activity in various cell lines is presented in Table 3.

### DOX-ERLP showed enhanced *in vivo* antitumor efficacy in H22 bearing mice

**Figure 7:**
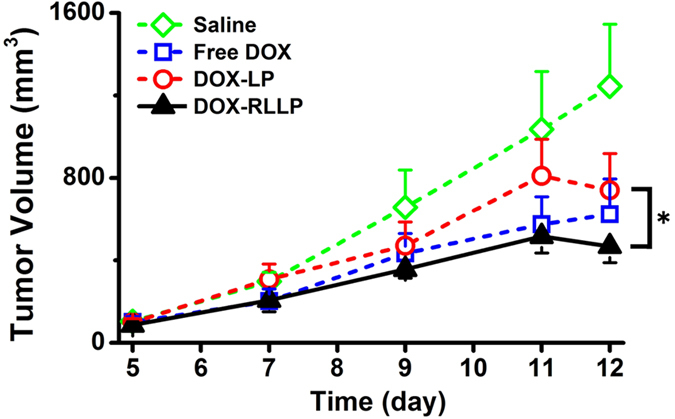
Tumor volume changes in ICR mice treated with N.S. control (◊; green), DOX-LP (⚪; red), Free DOX (□; blue), and DOX-ERLP (▲; black) (*p < 0.05).

Because DOX showed potent inhibition of H22 cancer cells, we did an efficacy comparison in the H22 xenograph model in mice^29^. After dosing 4 groups (8 mice in each) of the H22 tumor bearing mice 4 times, once every other days with normal saline (Group A), Free DOX (Group B) DOX-LP (Group C) or DOX-ERLP (Group D) containing 5 mg/kg DOX, mouse death was observed in the normal saline group so the experiment was ended. During the treatment, the tumor growth was followed by size measurement. Shown in Fig. 7, tumors in the normal saline group (green lines) grow fast. In comparison with normal saline group on day 12th, DOX-LP demonstrated growth inhibition (red lines) of 44%, free DOX (blue line) of 46%, and the DOX-ERLP (black) of 68%. Statistical analysis showed a p value < 0.05 for all groups.

**Figure 8:**
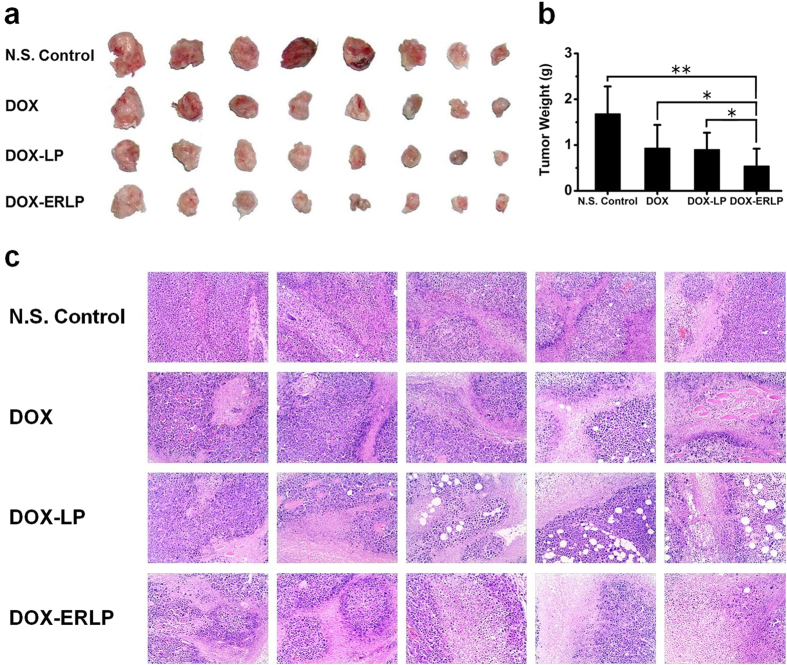
The *in vivo* efficacy comparison in 4 different groups with 8 mice in each group: DOX-ERLP significantly reduced tumor size and increased necrotic area within tumors after 4 injections of N.S. control, free DOX solution, DOX-LP and DOX-ERLP (n = 8). (**a**) Picture of end point tumors, (**b**) Tumor weight, (**c**) HE stain of tumor slices to identify the necrosis area.

The tumor size reduction was also confirmed by the end point tumor weight measurement (Fig. 8a,b), and the tumor tissue damage was evaluated by HE stain (Fig. 8c). In comparison with the saline group, DOX-ERLP treatment inhibited an average of 68% tumor growth by weight. Also, HE staining was performed to evaluate the necrotic region. The normal tissues show a clear tumor cell structure and hyperchromatic nucleus, whereas the necrotic region displays relative faint color with no clear cellular structure. In each group, necrotic regions were observed to various levels. The saline group showed an estimated necrotic level of 5–30%; increased levels of necrotic areas were seen with DOX and DOX-LP treatment; the most dramatic amount of necrosis (estimated 45–80%) was observed in DOX-ERLP treatment. Taken together, 4 injections of DOX-ERLP significantly reduced tumor size by 68% and caused 45–80% necrosis within the tumor.

### Conclusion and Prospects

DOX is a natural product cancer drug with strongly negative side effects. Various chemical modifications of DOX^29^,^30^ aiming to enhance tumor targeting as well as liposomal formulations have been tried to improve its antitumor activity and lowering its side effects. However, our preliminary results demonstrated that DOX-LP was not absorbed rapidly by MCF7 cells and showed no improved efficacy towards the DOX-resistant strain (MCF7-adr). Addition of a cationic polymer (Eudragit RL100) to DOX liposomes resulted in novel composite nanoparticles (DOX-ERLP) which are much more effective than the DOX or DOX-LP. Maybe the cationic charge on ER provided better interaction of the DOX-ERLP liposome with the partially negatively charged cell membranes, which led to the improved uptake rate of the liposome and better therapeutic effect.

In comparison to DOX-LP, DOX-ERLP demonstrated uniform particle sizes of approximately 200 nm, slower DOX release, higher cell absorption, and significantly improved *in vitro* and *in vivo* efficacy to multiple cancer cell lines including MCF7/adr. Our work represents the first use of the cationic FDA approved pharmaceutic adjuvant, Eudragit RL100 polymer, in liposome modification, which dramatically improved cellular uptake of the DOX-loaded liposomes and antitumor activity. Eudragit RL100 polymer modification is well-tolerated in biological system when being injected intravenously; this indicates that Polymethacrylate derivatives might be valuable additives for liposomes that are suitable for cancer drug delivery.

## Additional Information

**How to cite this article**: Wang, W. *et al*. Cationic Polymethacrylate-Modified Liposomes Significantly Enhanced Doxorubicin Delivery and Antitumor Activity. *Sci. Rep.* **7**, 43036; doi: 10.1038/srep43036 (2017).

**Publisher's note:** Springer Nature remains neutral with regard to jurisdictional claims in published maps and institutional affiliations.

## Figures and Tables

**Table 1:** The size and potential of various polymethacrylate modified liposomes.

ER:LP ratio	0:1	1:12	1:6	1:3	1:1	2:1	1:0
Zavd (nm)	408	255.4	204.3	159.1	189.5	178.2	84.02
PdI	0.722	1.000	0.598	0.399	0.127	0.141	0.100
Imd (nm)	1643	817.4	599.2	252.3	217.0	208.6	94.30
Vmd (nm)	2502	654.9	466.0	166.2	215.1	203.4	73.09
Nmd (nm)	112	30.83	33.96	45.33	146.3	133.3	56.51
ZP (mV)	−2.20	35.4	33.5	40.8	40.1	48.3	55.1

**Table 2:** DOX level in cells and in supernatants.

Vesicles	Flow cytometry assay		Florescence in cell pellets (Unit)		Florescence in Supernatant (Unit) 100%	Total DOX (Unit) 100%
	Florescent cells (%)	DOX Florescence (Unit)	Unwashed 50%	Washed 50%		
Free DOX	81	53	1437	1383	4347	71280
DOX-LP	83	209	4507	4259	16842	72210
DOX-ERLP	77	406	7394	7120	37074	71500

**Table 3:** IC_50_ of DOX in different vehicles to MCF7, MCF7/adr and H22 cells (n = 3).

Vesicles	IC_50_		
	MCF7 (μg/ml)	MCF7/adr (μg/ml)	H22 (nM)
Free DOX	0.90 ± 0.05	13.90 ± 3.55	15 ± 1
DOX-LP	0.91 ± 0.32	24.07 ± 2.18^*b^	12 ± 1
DOX-ERLP	0.63 ± 0.10^*a^	6.72 ± 2.53^*c,**d^	6 ± 0.5^**e,**f^

P < 0.05: *P < 0.01: ** ^a^P < 0.05, DOX- solution vs DOX-ERLP; ^c^P < 0.05, DOX- solution vs DOX-LP; ^c^P < 0.05, DOX- solution vs DOX-ERLP; ^d^P < 0.01, DOX-LP vs DOX- ERLP; ^e^P < 0.01, DOX- solution vs DOX-ERLP; ^f^P < 0.01, DOX-LP vs DOX- ERLP.
